# Compositional, ultrastructural and nanotechnological characterization of the SMA strain of *Saccharomyces pastorianus*: Towards a more complete fermentation yeast cell analysis

**DOI:** 10.1371/journal.pone.0200552

**Published:** 2018-07-11

**Authors:** Greg Potter, Chantel W. Swart, Pieter W. J. van Wyk, Mart-Mari Duvenhage, Elizabeth Coetsee, Hendrik C. Swart, Suzanne M. Budge, R. Alex Speers

**Affiliations:** 1 Canadian Institute of Fermentation Technology, Department of Process Engineering and Applied Science, Dalhousie University, Halifax, NS, Canada; 2 Centre for Microscopy, Faculty Natural and Agricultural Sciences, University of the Free State, Bloemfontein, South Africa; 3 Department of Physics, University of the Free Sate, Bloemfontein, South Africa; 4 Department of Process Engineering and Applied Science, Dalhousie University, Halifax, NS, Canada; College of Agricultural Sciences, UNITED STATES

## Abstract

Nano scanning Auger microscopy (NanoSAM) and time-of-flight secondary ion mass spectrometry (TOF-SIMS) have been used in materials science research for some time, but NanoSAM, in particular, has only recently been applied to biological specimens. Here, the first concurrent utilization of NanoSAM, TOF-SIMS and microscopic techniques for the examination of a standard beverage fermentation strain of *Saccharomyces pastorianus* uncovered the presence of intracellular networks of CO_2_ in fermenting cells. Respiring cells produced few bubbles and instead had large internal vacuolar structures. Transmission electron microscopy analysis also showed osmiophilic layers at the cell exterior of fermenting cells that became more prevalent with fermentation duration, while osmiophilic layers were largely absent in respiring cells. TOF-SIMS analysis showed a compositional difference at the exterior and interior of SMA cells and between fermenting and respiring cells. Fermenting cells also appeared to have different 3-OH oxylipin profiles compared to respiring cells based upon examination with immunofluorescence microscopy. The results of this work and further study using these materials science techniques will substantially enhance our understanding of the chemical, ultrastructural and metabolic changes that occur in fermentation yeasts.

## Introduction

A key tenet of materials science is that a comprehensive compositional survey of a substance is only possible when surface analysis is combined with depth profiling. Fortunately, the field has developed several nanotechnological instruments to achieve such a complete approach including both Nano scanning Auger microscopy (NanoSAM) and time-of-flight secondary ion mass spectrometry (TOF-SIMS). NanoSAM is a technique which combines the imaging capacity of scanning electron microscopy (SEM), the elemental analysis capability of Auger electron spectroscopy (AES) and depth profiling conferred by the etching functionality of an Argon (Ar^+^) gun. Recently, NanoSAM was applied to biological specimens for the first time in a new application called Auger-architectomics [1]. Auger-architectomics, while still a relatively novel technique in biological sciences, has proven immensely useful in diverse fields such as translational medicine, cancer biology and fermentation science [2–4]. In yeast and filamentous fungi biology, several different strains, including *Nadsonia fulvescens* [1], *Cryptococcus curvatus* [5] and the fermentation strains *Saccharomyces cerevisiae* CBS 1171 NT and *Saccharomyces pastorianus* WS 34–70 [2,3], have already been characterized using this nanotechnology.

Even though it is well-known that CO_2_ and ethanol are released as by-products during yeast fermentations, CO_2_ was never observed in yeast cells until the application of Auger-architectomics indicated the presence of intracellular CO_2_ gas bubbles [2,3]. This discovery represented a paradigm shift in current models of intracellular gas generation, transport and cellular metabolism. Interestingly, during Auger-architectomal analysis, it was also shown that the presence of gas bubbles inside cells deformed and compressed internal organelles [3]. With this finding, it has now become paramount to examine how gas bubble formation effects the metabolism, performance and vitality of fermenting yeasts. Since 3-OH oxylipins are presumed to play a significant role in flocculation during fermentation [6–8], the effect of gas bubble formation and thus, fermentation, on 3-OH oxylipin production is also of interest. Furthermore, additional fermenting yeasts, including standard test strains, must be studied with Auger-architectomics and TOF-SIMS to understand the variability between strains in terms of bubble generation, deformation of cell ultrastructure and cellular composition.

Currently, the SMA strain of *S*. *pastorianus* has become a standard test strain as it is the strain required in the America Society of Brewing Chemists miniature fermentation assay [9]. The SMA strain has been characterized in terms of its flocculation behaviour and growth kinetics [9,10], and has proven to be useful in studies on wort fermentability [11] and premature yeast flocculation [12]. Coincident changes in fatty acid profile, flocculation and cell surface hydrophobicity have also been studied in the SMA strain during growth in the miniature fermentation assay [8]. Furthermore, the SMA strain of *S*. *pastorianus* has also been shown to produce the potentially bioactive oxylipins, 3-OH 8:0 and 3-OH 10:0, when grown in lab-scale fermentations [8]. Thus, the SMA strain is well characterized using classical methods and an appropriate candidate for further analysis of its cellular structure and composition using nanotechniques.

In this study we have analyzed the standard SMA strain of *S*. *pastorianus* with Auger-architectomics and TOF-SIMS (i) to further demonstrate the nascent biological applications of these techniques, (ii) to investigate the influence of bubble formation on cellular composition in fermenting yeasts and (iii) to examine the effects of fermentative growth and bubble formation on 3-OH oxylipin production. To achieve this, we have coupled nanotechnological analyses of the SMA strain using Auger-architectomics (or NanoSAM) and TOF-SIMS with microscopic examination via light microscopy (LM), transmission electron microscopy (TEM), high resolution (Hi-Res) SEM and immunofluorescence with confocal laser scanning microscopy (CLSM). For the purposes of comparison, all analyses were jointly conducted on fermenting and respiring yeast cells.

## Materials and methods

### Cultivation and analysis

The SMA strain of *S*. *pastorianus* (obtained from VLB Berlin, Biological Laboratory, Seestraße 13, D-13353 Berlin) was streaked from an agar slant onto a yeast malt (YM) agar plate and cultivated for 48 h at 29°C. A pre-inoculum was then prepared by inoculating a loopful of the fresh cells into 250 mL Erlenmeyer flasks containing 100 mL of fermentable and non-fermentable media and incubating the flasks at 29°C for 24 h (160 rpm). The fermentable media was glucose YM broth consisting of 10 g/L glucose, 3 g/L yeast extract, 3 g/L malt extract and 5 g/L peptone while the non-fermentable media was yeast peptone glycerol (YPG) broth containing 30 mL/L glycerol, 10 g/L yeast extract and 20 g/L peptone. After 24 h, 1 mL of the respective pre-inoculums were transferred to additional 250 mL flasks containing the same fermentable and non-fermentable media, and the flasks were incubated using the previously described conditions. Sufficient flasks were prepared so that there were 3 biological replicates at each sampling time. At each of 24, 48 and 72 h, cells were collected from the 3 replicates of both growth mediums and were analyzed using LM, NanoSAM, Hi-Res SEM, TEM, TOF-SIMS and CLSM.

### Light microscopy

The cells were viewed using a light microscope (Axioplan, Zeiss, Germany) with coupling to a Colourview Soft Digital Imaging System (Münster, Germany) at 1000 X magnification under oil immersion to check the cells for granularity and also to ensure the purity of the cultures. A granular appearance was expected in the fermenting cells since intracellular gas bubbles appear as light scattering granules.

### Nano scanning Auger microscopy

Cells were prepared according to Swart et al. [1] and Kock et al. [13]. At each sample collection time, 5 mL culture volumes of the fermenting cells (YM broth) were collected. Since cells grown in YPG broth (respiring) grow at a decreased rate, 15 mL volumes were collected from this media in order to obtain a pellet of sufficient mass for further analyses. Thereafter, the culture volumes were centrifuged at 1450 g for 5 min and the pellets were double-washed with sterile distilled water. The pellets were then fixed for at least 2 h at room temperature in 0.1 M (pH 7) sodium phosphate-buffered 3% glutaraldehyde and subsequently for 1 h in 1% osmium tetroxide buffered in the same solution. Between each fixation step the cells were rinsed with the same buffer solution (once after the glutaraldehyde step and twice after the osmium tetraoxide step).

The cells were then dehydrated by sequential exposure to 50, 75 and 95% ethanol for 20 minutes at each concentration and were subjected to two final 100% ethanol steps, each lasting for 1 h. Cells were dried into powder form using a critical point drier, after which the cells were mounted on stubs and sputter coated with gold to make them electron conductive. Samples were then examined with the NanoSAM which consisted of a PHI 700 Nanoprobe (Physical Electronics, Inc. Japan) equipped with SEM and scanning Auger microscopy capabilities. For SEM and scanning Auger microscopy analyses the field emission electron gun was set as follows: 2.29 A filament current, 3.58 kV extractor voltage and 226 μA extractor current. At these settings, a 25 kV and 10 nA electron beam with a diameter of 12 nm was produced to facilitate the Auger analyses and SEM imaging. The upper pressure of the ultra high vacuum was 9.9E-10 Torr and the pressure of the main chamber was 3.47E-10 Torr. 6 nm SE image resolution at the analysis position and 8 nm Auger resolution were achieved, while 1–10 nm information depth and a 1–0.1% detection limits were possible [14]. To obtain the Auger point analyses, 10 cycles per survey, 1 eV per step and 50 ms per step were used. The Ar^+^ ion sputtering gun was set as follows: 2 kV beam voltage, 1.5 μA ion beam current with a 1 mm x 1 mm raster area, which produced a sputter rate of 15 nm/min. An alternating sputter mode with sputter intervals of 1 min and sputter time of 2 min was used without any rotation. The % atomic relative concentrations of carbon (C), gold (Au), osmium (Os), nitrogen (N), oxygen (O), phosphorus (P) and sulfur (S) were monitored during AES analysis. Relative sensitivity factors were used to calculate the concentration of elements above atomic number 3.

### High-resolution scanning electron microscopy

The cells that were harvested, prepared, analyzed and Ar^+^ etched in the NanoSAM were also examined with a Hi-Res SEM in order to obtain high resolution images of intracellular ultrastructure. This instrument was a JEOL JSM-7800F Extreme-resolution Analytical Field Emission microscope (USA) fitted with a lower electron detector at the following settings: 5 kV and 9.634E-5 Torr.

### Transmission electron microscopy

Cells were prepared according to Swart et al. [1]. Cells for TEM analysis were harvested, washed and fixed in the same manner as the NanoSAM analyzed cells. Thereafter, the cells were dried by sequential exposure to 50, 75 and 95% acetone for 20 min at each concentration and were subjected to two final 100% acetone steps, each lasting for 1 h. Once dehydrated, the cells were embedded in epoxy resin which was polymerized at 70°C for 1 h. The embedded cells were then cut into ultra-thin 60 nm sections using a Leica Ultracut UM7 microtome that was fitted with a glass knife. A double staining was then applied to all sections, first with uranyl acetate for 3 min and then with lead citrate for 10 min. All sections were viewed using TEM [FEI (Phillips) CM 100, Netherlands].

### Time-of-flight secondary ion mass spectrometry

Cells for TOF-SIMS analysis were harvested and prepared in the same manner as cells examined with the NanoSAM, except they were not coated with gold and they were mounted on copper stubs with double-sided tape. TOF-SIMS mass spectra and ion maps were obtained with a secondary ion mass spectrometer TOF-SIMS V (ION-TOF GmbH, Münster, Germany) fitted with a high mass resolution (>10 000) time-of-flight analyzer. Secondary ion mass spectra were recorded from clumps of cells in an approximately 20 μm × 20 μm area of the sample. During measurement the analyzed area was irradiated with pulses of 30 keV Bi^1 +^ ions from a primary ion gun at a 10 kHz repetition rate and a flood gun (low-energy electrons) was used to compensate for any surface charging. A 2.5 kV argon cluster gun was also used to sputter the samples in a 100 μm x 100 μm region to allow for depth profiling analysis. Mass spectra were recorded from m/z 0–200 and intensities of the negative ions C^-^, NH^-^, O^-^, OH^-^, P^-^, and S^-^ were monitored. Intensities were normalized based on total intensity at each sputtering time to allow for comparison between samples, to account for any operational variability between runs [15] and to negate any difference in the amount of cell material between samples.

### Confocal laser scanning microscopy

Cells for CLSM were cultivated in the same manner as cells for NanoSAM analysis. At each harvesting time, 400 μL of cell culture from YM media and a pellet from a concentrated 10 mL aliquot of cell culture in YPG media were transferred to 2 mL microcentrifuge tubes and washed in 400 μL pH 6.5 phosphate buffered saline PBS. Thereafter, the cells were incubated with 30 μL rabbit-raised 3-OH oxylipin-specific antibodies (1:10 dilution v/v) for 1 h in the dark [16]. Unbound primary antibody was then removed by centrifugation and a wash. A 30 μL aliquot of the fluorescein isothiocyanate-conjugated secondary antibody (1:10 dilution v/v) was subsequently added to the pellet and a second 1 h dark incubation followed. The secondary antibody was then removed by washing and the slide and cover-slip mounted cells were viewed with a Nikon TE2000-E inverted Confocal C1 microscope (Tokyo, Japan), equipped with a 200 mW Argon-ion 454–676 nm excitation laser (Spectra Physics, Santa Clara, CA 95054, USA).

## Results

### Light microscopy

**Fig 1:**
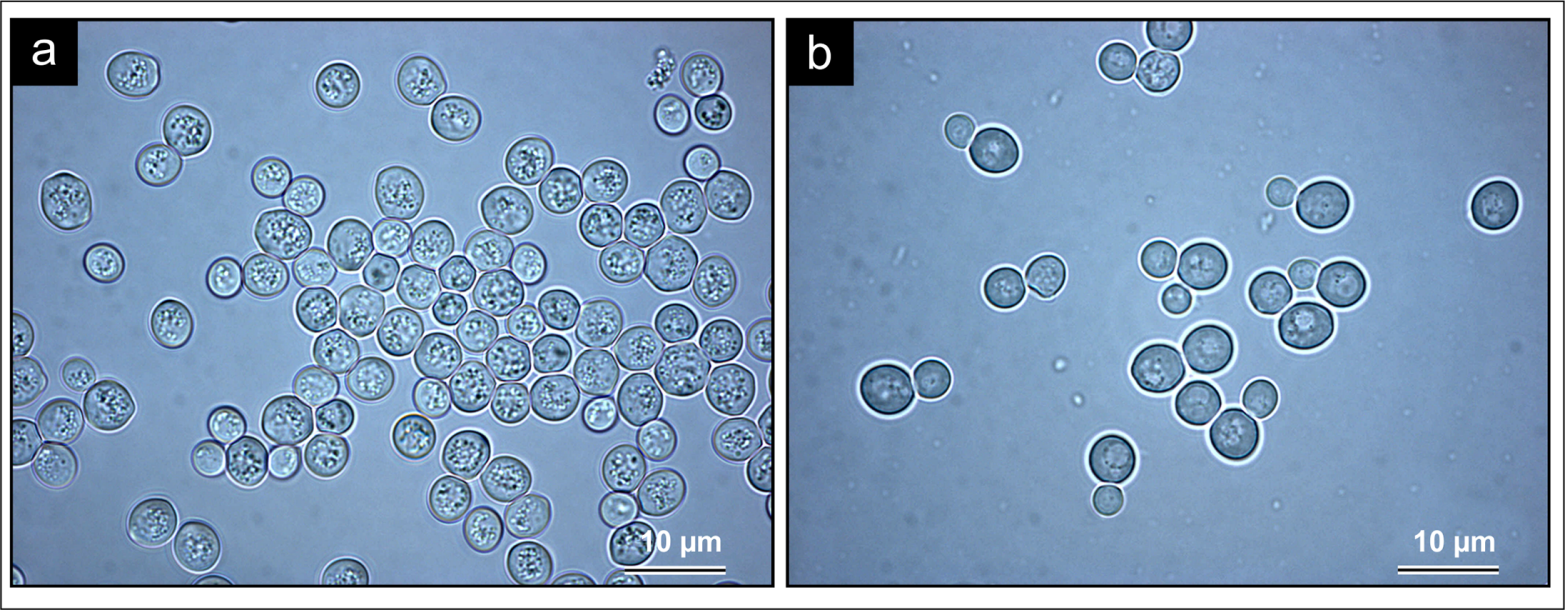
LM analysis of the SMA strain of *Saccharomyces pastorianus* grown in YM and YPG media for 72 h. (A) LM analysis (1000 X) of cells grown in YM media for 72 h. A distinct granular appearance of the cells is evident. (B) LM analysis (1000 X) of cells grown in YPG for 72 h. The cells were less granular and had distinct vacuolar structures.

The SMA strain showed gas bubbles as light scattering granules inside the cells when grown in YM (fermentable) media (Fig 1A). There was little to no light scattering granules inside the cells grown in YPG (non-fermentable) media (Fig 1B). Cells grown in YPG media also tended to have large vacuolar structures when compared to cells grown in YM media. In YM-grown cells, across the population, a granular appearance (suggesting gas bubble presence) was observed in both mother and daughter cells. The population-wide granularity also increased successively over the course of the fermentation (24 h– 72 h). Cells grown in YM were often misshapen, whereas cells grown in YPG maintained a more ovoid shape.

### Nano scanning Auger microscopy and hi-res scanning electron microscopy

**Fig 2:**
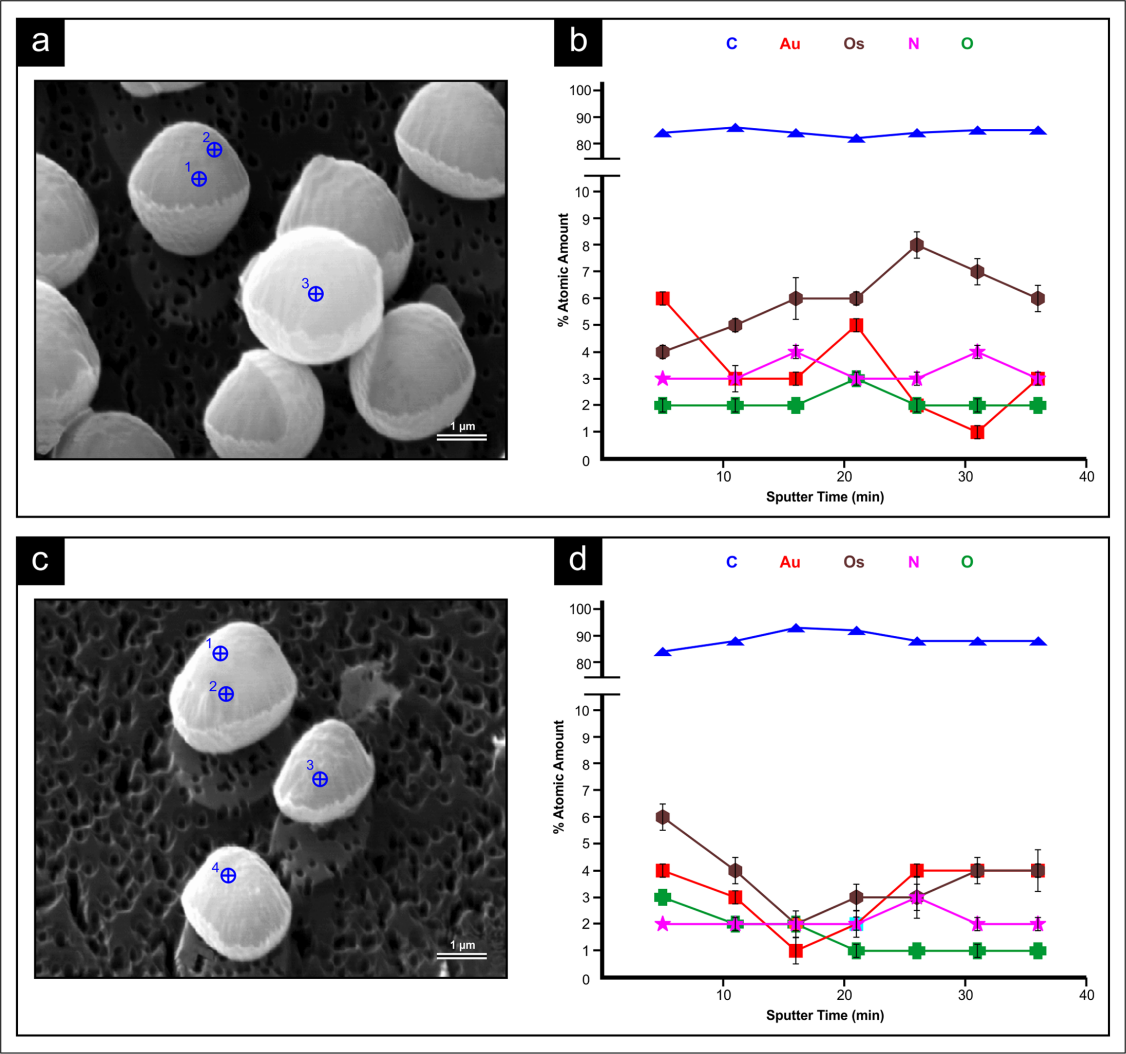
NanoSAM SEM and AES analysis of the SMA strain of *Saccharomyces pastorianus* grown in YM and YPG media for 24 h. SEM micrograph of (A) YM 24 h cells and (C) YPG 24 h cells. The targets for elemental analysis are shown by the blue cross circles. Graphs of elemental analysis as the mean % relative atomic concentration and sputtering time from YM 24 h cells (B) and from YPG 24 h cells (D). Values from targets 1 and 2 were used for the YM and YPG cells.

Targeted spot analysis (Fig 2A and 2C) in all samples from both YM and YPG media revealed the cells were composed primarily of carbon, ranging from 82–96%, while Au, Os, N and O were present in all samples in smaller but similar amounts (Fig 2B and 2D). Percent relative atomic concentrations of C, N, O, Au and Os varied with sputtering time (Fig 2B and 2D) from the same defined location in the cell at depths up to 540 nm. P and S were also monitored in an attempt to differentiate between protein and lipid-rich regions in the cell as most proteins contain sulfur but not phosphorus and because phosphorus analysis can be used for lipid quantification [17, 18]. Unfortunately, neither P nor S were detectable. To compare the differences in the composition of fermenting (YM) and non-fermenting (YPG) conditions, an unpaired t-test for the level of each element at each sputtering time from 2 spot analyses (target 1,2) on a single non-fermentative (YPG) grown cell and from 2 spot analyses (target 1,2) on a single fermentative (YM) grown cell (Fig 2A and 2C) was employed. The differences in % atomic amount of each element (C, Au, Os, N, O) across sputtering times from the single fermentative and non-fermentative grown cell were not statistically significant.

**Fig 3:**
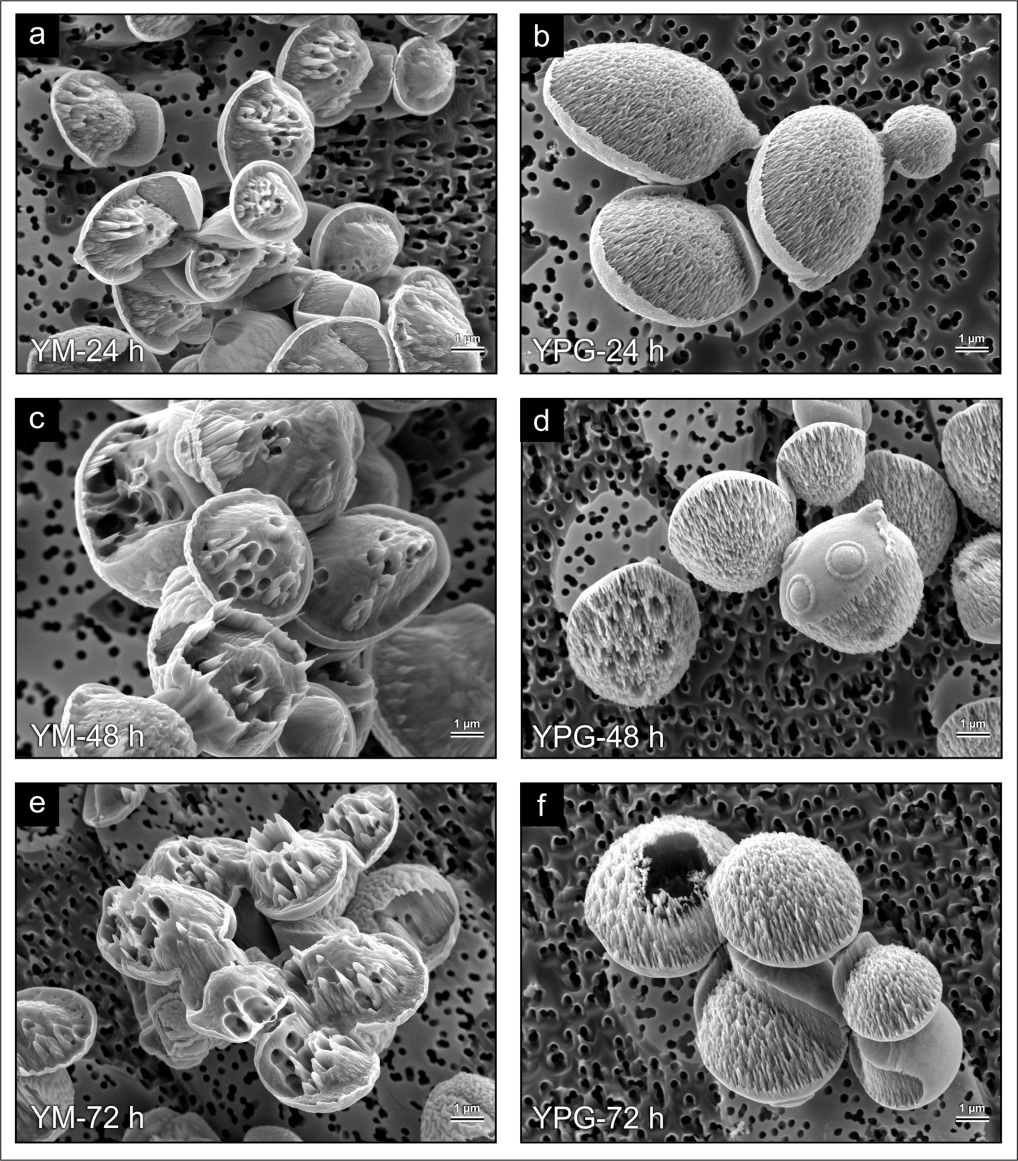
Post-Ar^+^ etching (36 mins) Hi-Res SEM analysis of the SMA strain of *Saccharomyces pastorianus* grown in YM and YPG media. For the 24 h (A), 48 h (C) and 72 h (E) cultures grown in YM media, the network of bubbles became more complex as the fermentation progressed. The 24 h (B), 48 h (D) and 72 h (F) cultures grown in YPG media lacked a network of bubbles, but certain cells contained large holes left by vacuolar structures.

After etching, cells were viewed with Hi-Res SEM to obtain high quality images of the gas bubbles and larger vacuolar structures inside the cells grown in YM and YPG media, respectively. A similar trend was observed in the YM-grown cells with an increase in the number of gas bubbles (shown as defined, circular intracellular indentations) with fermentation time (24 h– 72 h) (Fig 3A, 3C and 3E). This corresponds well with that observed with LM. The size and complexity of the bubble networks within the YM-grown cells also increased with fermentation duration; however, there was a non-homogenous distribution of bubble networks between individual cells within a field of view. Cells from the 72 h culture cultivated in YM media appeared to have a more wrinkled cell exterior (Fig 3E). Consistent with the LM results obtained, the YPG-grown cells contained larger vacuolar structures (Fig 3F) and no or very few bubbles (e.g. defined, circular intracellular indentations) could be observed at any sampling time (Fig 3B, 3D and 3F).

### Transmission electron microscopy

**Fig 4:**
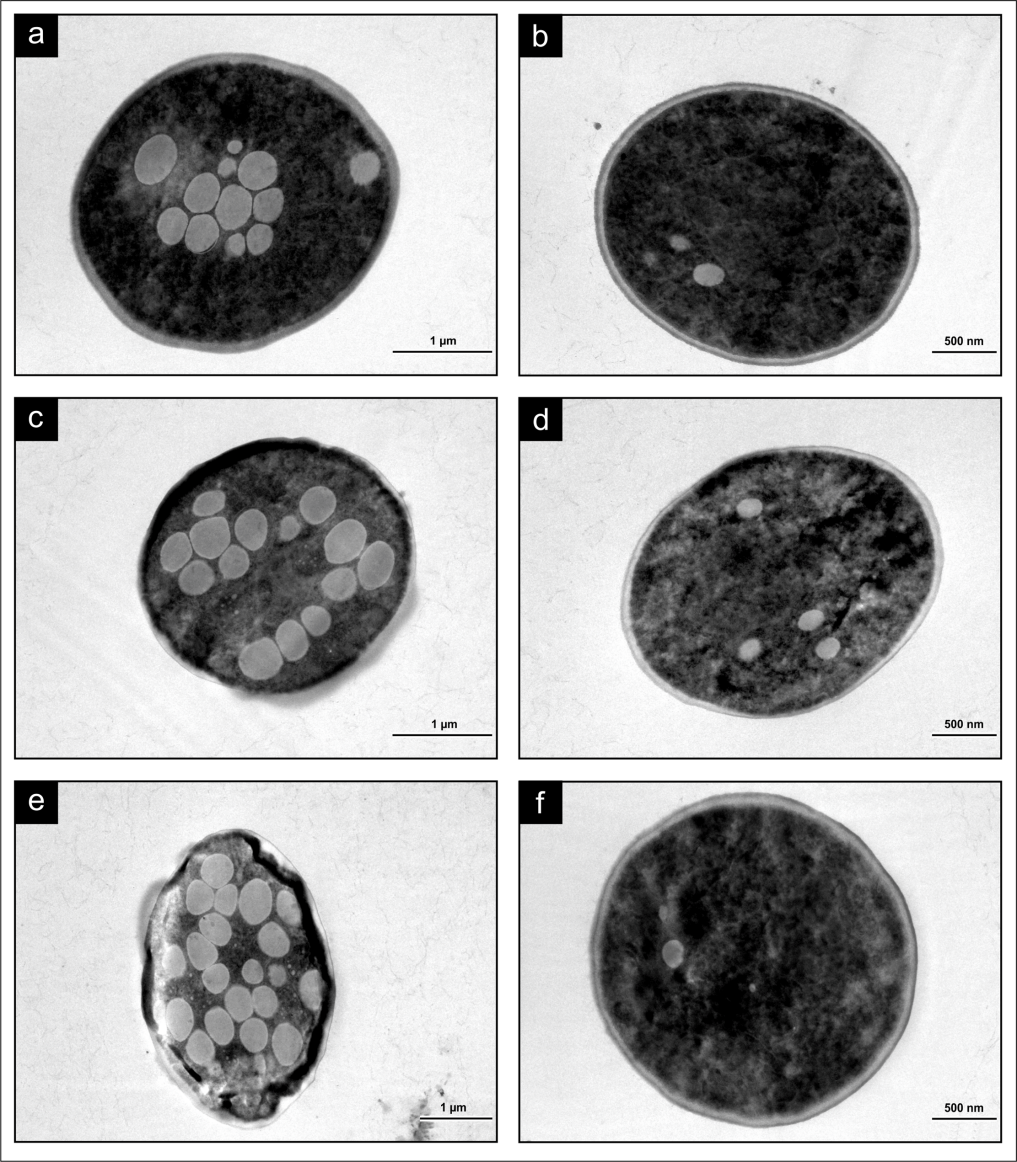
TEM analysis of the SMA strain of *Saccharomyces pastorianus* grown in YM and YPG media. Cultures grown for 24 h (A), 48 h (C) and 72 h (E) in YM media demonstrated the increase in number of bubbles and change in cell morphology that occurred as fermentation progressed. Cultures grown for 24 h (B), 48 h (D) and 72 h (F) in YPG media showed diminished bubble formation.

To verify the results obtained with LM and NanoSAM (including Hi-Res SEM imaging), cells grown in both fermentable (YM) and non-fermentable (YPG) media were subjected to TEM analysis. Results obtained indicate a number of electron-transparent gas bubbles inside the fermenting cells that increase with fermentation time to a point where the 72 h aged cells were almost completely filled (Fig 4A, 4C and 4E). The intracellular structures were not enveloped by a membrane suggesting that they were indeed bubbles and not cell organelles. Cells grown in non-fermentable media contained few electron-transparent gas bubbles (Fig 4B, 4D and 4F). These results coincide with observations using LM, NanoSAM and Hi-Res SEM. Cells grown in YM media also developed more pronounced dark-staining, osmiophilic layers at the cell exterior as the fermentation progressed (Fig 4A, 4C and 4E). Osmium textroxide, in addition to functioning as a fixative, also serves as a lipid specific stain that turns tissues rich in unsaturated lipid, in particular, dark in colour [19]. No such osmiophilic layers could be observed at the exterior of any of the YPG-grown cells (Fig 4B, 4D and 4F). Cells grown in YM media also developed more wrinkles at the cell exterior over the fermentation course (Fig 4A, 4C and 4E).

### Time-of-flight secondary ion mass spectrometry and confocal laser scanning microscopy

**Fig 5:**
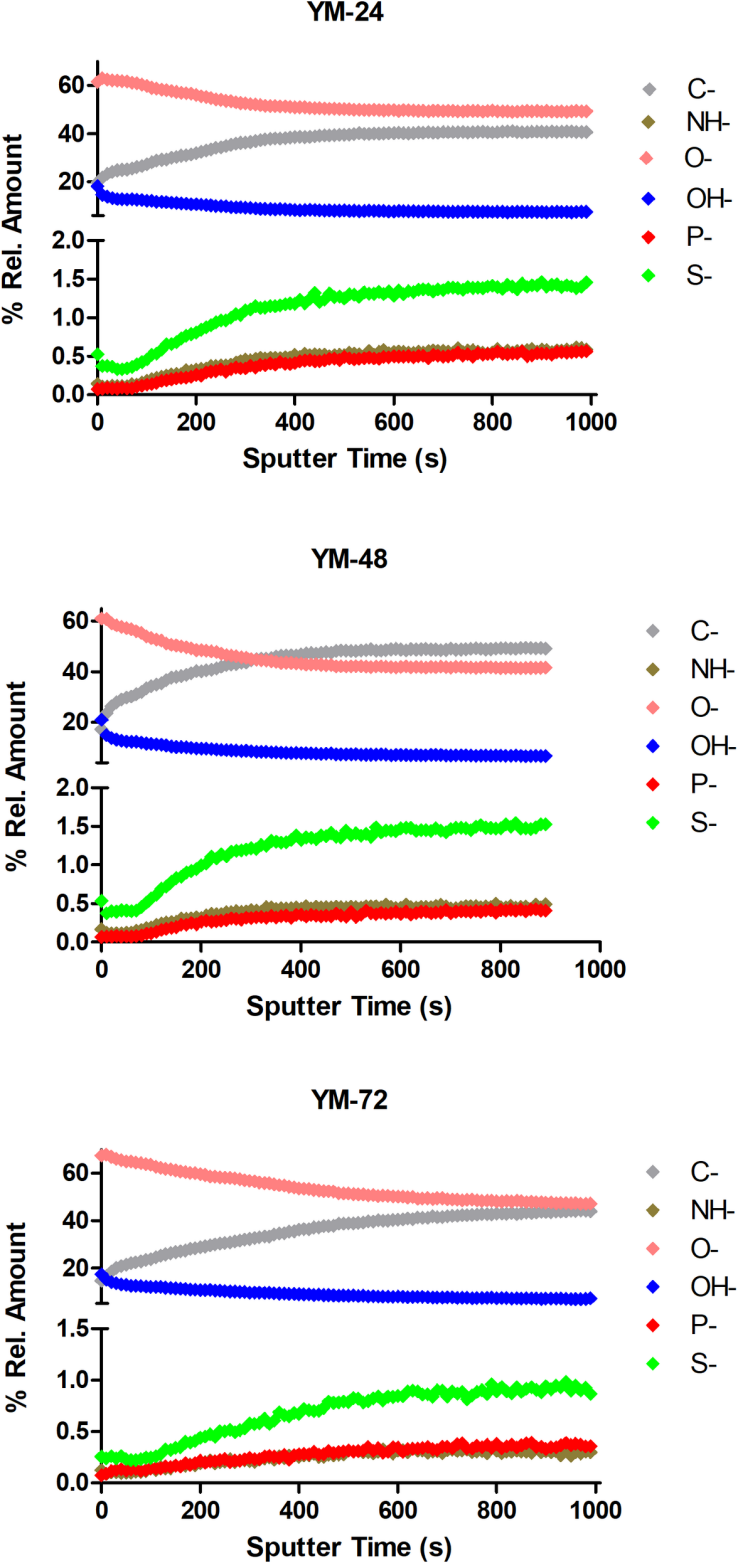
TOF-SIMS depth profiling analysis of the SMA strain of *Saccharomyces pastorianus* grown in the fermentative glucose-containing YM media for 24 h, 48 h and 72 h. The negative atomic ions C^-^, NH^-^, O^-^, OH^-^, P^-^ and S^-^ were monitored. The intensities were normalized at each sputtering time and are expressed as % relative intensity.

**Fig 6:**
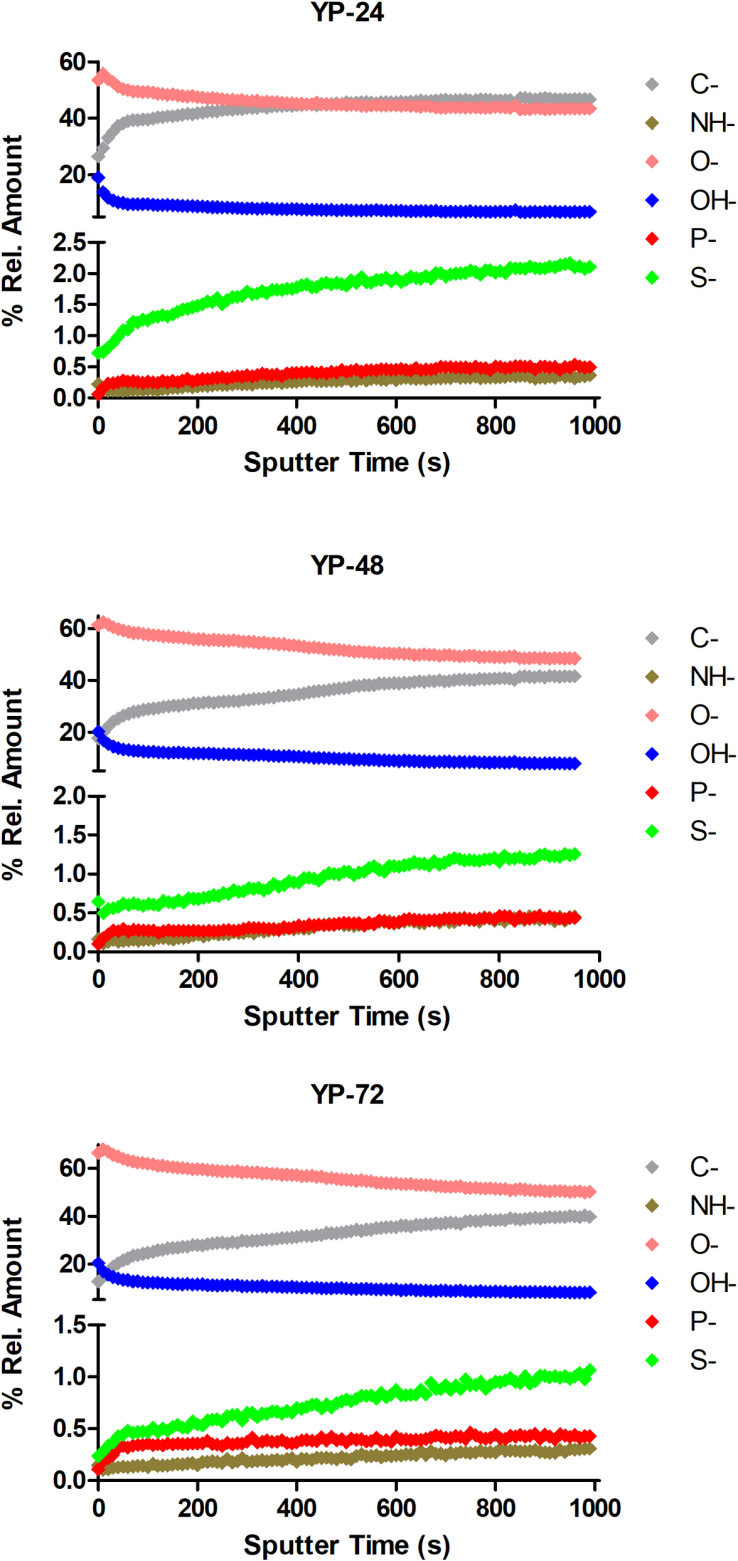
TOF-SIMS depth profiling analysis of the SMA strain of *Saccharomyces pastorianus* grown in the non-fermentative glycerol-containing YPG media for 24 h, 48 h and 72 h. The negative atomic ions C^-^, NH^-^, O^-^, OH^-^, P^-^ and S^-^ were monitored. The intensities were normalized at each sputtering time and are expressed as % relative amounts.

TOF-SIMS depth profiling analysis showed that the cell compositions changed with sputtering time (Fig 5; Fig 6), and thus, appeared to differ at the exterior and interior of the cell. In cell clumps from both YM and YPG-grown cultures, C^-^ and O^-^ were the most intense ions and these converged as sputtering time increased. In particular, C^-^ levels were most intense and O^-^ were the least intense in 48 h fermenting cells. Respiring cells had the highest intensity C^-^ ions at 24 h that decreased over time, while O^-^ ion intensity increased with growth time to elevated levels at 72 h. TOF-SIMS analysis also indicated P^-^ and S^-^ could be detected in both cell types, with greater intensity S^-^ than P^-^ ions.

**Fig 7:**
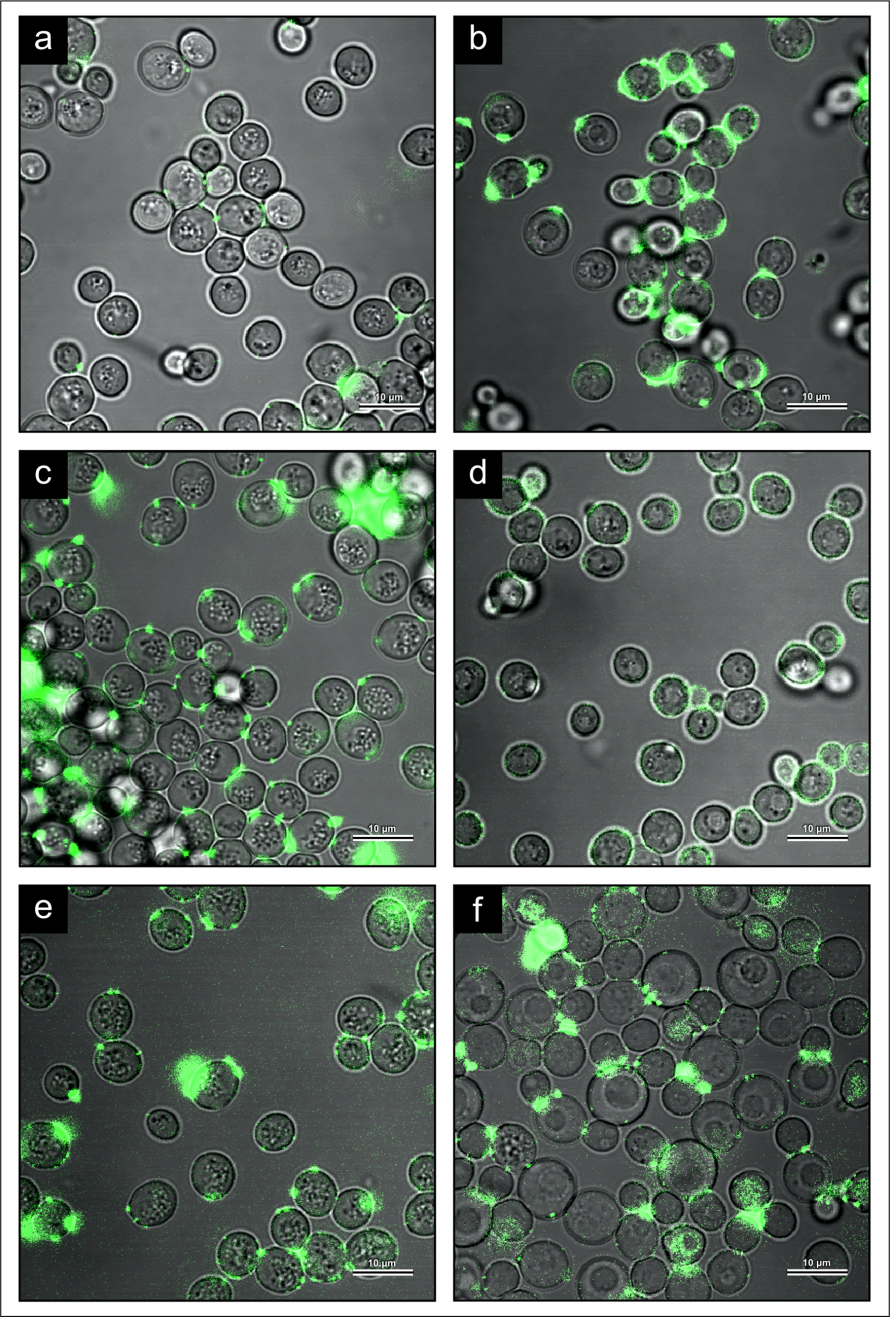
CLSM analysis of the SMA strain of *Saccharomyces pastorianus* grown in YM and YPG media for 24 h, 48 h and 72 h. 3-OH oxylipin level increased from 24 h (A), 48 h (C) and 72 h (E) during growth in YM media. In YPG media, there also appeared to be more 3-OH oxylipins after 72 h (F) relative to 24 h (B) and 48 h (D).

CSLM using immunofluorescence labelling showed that under fermentation growth conditions, the SMA strain produced progressively more 3-OH oxylipins as fermentation time increased from 24 to 72 h (Fig 7A, 7C and 7E). After 24 h growth time, the respiring cells produced more 3-OH oxylipins (Fig 7B) as compared to fermenting cells of the same age (Fig 7A). However, at 72 h, more 3-OH oxylipins seemed to be present in fermenting (YM) cells than in respiring (YPG) cells (Fig 7E and 7F). The localization and distribution of fluorescence in antibody labelled fermenting cells was compared to respiring cells to determine the influence of bubble formation on 3-OH oxylipin profile. Unfortunately, there was not a clear indication that 3-OH oxylipins localize differently in fermenting or respiring yeasts. However, some fermenting cells within a field of view did fluoresce more brightly than others, which aligned with the Hi-Res SEM results of the fermenting cells where certain cells appeared more bubble-filled.

## Discussions

### Light microscopy

LM results for the SMA strain correspond to those of Swart and co-workers [2], where gas bubbles were observed as light scattering granules inside YM-grown cells, and YPG-grown cells produced very few light scattering granules. Swart and co-workers [2] also noted bubble production as intracellular granules in both older and younger cells, which may suggest that bubble production, and thus fermentation, is not strictly linked to cell age. The overall shape of the YM-grown cells proved to be another unexpected result. As the discovery of intracellular gas bubbles inside fermenting yeasts is a recent finding, there have been no coincident examinations of CO_2_ bubble formation and other cellular processes. However, given the effect of bubble formation on the overall shape of the cell, it seems plausible that normal organelle and cell function would be influenced/affected. Indeed, intracellular gas accumulation in other biological systems has been shown to have profound physiological effects, including control of cell proliferation, induction of apoptosis, and relaxation and constriction of vascular tissues [20,21].

### Nano scanning Auger microscopy and hi-res scanning electron microscopy

In the first applications of NanoSAM AES analysis to biological samples [1], elemental abundance of *Nadsonia fulvescens* cells was reported in terms of intensity. In reality, Auger electron intensities vary between elements and these variations should be taken into account to achieve measurement accuracy. Thus, the atomic concentrations values in this study derived using relative sensitivity factors represent more accurate measurements. The earlier NanoSAM AES analysis of *N*. *fulvescens* also revealed pronounced decreases in Os and Au intensities at 135 nm etching depths [1]. The presence of these elements in the samples are due to preparative steps for NanoSAM and SEM observation. A similar reduction in Os and Au % relative concentrations upon etching was not detected in any of the SMA cells grown in this study. While this discrepancy might be explained by variation in sample preparation, it may also point to differences in porosity and rigidity between biological samples. As vegetative brewing yeasts have a propensity to flocculate and are still metabolically active, it would seem that a less rigid cell exterior would be an asset. In contrast, a *N*. *fulvescens* ascosopre is likely more rigid as this is best suited for durability. The aforementioned Auger-architectomic study of *N*. *fulvescens* was also only able to detect trace amounts of N within the cells [1]. In the current study N was present in very similar levels as O, and was often more abundant than O (Fig 2B and 2D).

There are a number of reasons that the AES comparison of the non-fermentative and fermentative cells did not reveal data that were statistically significant. Firstly, the AES technique is only semi-quantitative [14]. Materials normally analyzed with AES are usually inorganic. Additionally, with Auger-architectomics, the biological samples must prepared by fixation, dehydration and sputter coating in gold. These steps are required to make the samples usable in the NanoSAM, but it is possible that the chemical and environmental exposures may have reduced the signal intensity of the AES analysis. Despite the lack of statistical significance, the use of the AES analysis here further demonstrates the nascent biological applications of this techniques. The atomic concentrations produced using relative sensitivity factors also represent an iterative development in the use of Auger-architectomics.

Hi-Res SEM investigations revealed that the size and complexity of bubble networks within a field of view varied. This suggests that the capacity for fermentation and bubble production could vary across the cell population. Heins et al. [22] reported highly dynamic subpopulation distributions at different growth stages when *Saccharomyces cerevisiae* was grown in a bioreactor and that these subpopulations changed in response to induced glucose and ethanol gradients. Speers et al. [23] also noted both flocculent and non-flocculent industrial brewing strains had non-normal surface charge distributions. It is possible that heterogeneity in bubble network size might arise from similar gradients or they may occur due to the natural non-synchronous cell populations that arise during fermentations [23–25]. Alternatively, some of the changes in cell ultrastructure could have been obscured during cell preparation or in the ultra-high vacuum environment of the NanoSAM. The Hi-Res SEM viewed cells were also more wrinkled later in the fermentation, which has been noted in previous SEM studies of older fermenting yeasts [26,27]. Another potential contributing factor to the wrinkled cell exteriors could have been the CO_2_ anaesthesia effect, where membrane domains in yeast are malformed by dissolution of molecular CO_2_ in the lipid bilayer [28].

### Transmission electron microscopy

When the YM-grown SMA strain was subjected to TEM analysis, the 72 h old cells (Fig 4E) were filled with gas bubbles to a greater extent than any other *S*. *cerevisiae* or *S*. *pastorianus* fermentation strains tested with this same TEM technique [2,3]. This observation emphasizes the variability between strains with respect to CO_2_ evolution, and identifies the SMA strain as an excellent candidate for future studies on bubble formation. These results also imply growth in YM media and TEM analysis could be a useful screening technique in selecting strains with high intracellular CO_2_ accumulation. Capture and sequestration of CO_2_ is a growing revenue source in beverage and fuel ethanol fermentation operations [29]. Thus, use of better techniques and model strains to understand how intracellular CO_2_ accumulation and release functions could make industrial CO_2_ sequestration more efficient.

Given the volume of bubbles present in the TEM-viewed cells, it is conceivable that the CO_2_ anaesthesia effect [28] contributed to the formation of the osmiophilic layers which were largest when bubble formation was most prevalent (Fig 4E). No such osmiophilic layers were present in the YPG-grown cells (Fig 4B, 4D and 4F), which warrants further investigation of the overall lipid content and fatty acid profile of fermenting vs. respiring cells. In earlier studies Kock et al. [6] noted the dark-staining, lipid-rich osmiophilic layers that were present in older fermenting cells, and described how these layers migrated through cells in a “ghost-like fashion”. This observation was made more than a decade before the discovery of intracellular gas bubbles in fermenting yeasts, but was likely indicative of their presence. It is now crucial to address how bubble formation and associated membrane changes during fermentation effect cell stability and flocculation. A wrinkled and rough exterior is thought to promote flocculation in fermenting yeasts [24]; however, bubble formation and flocculation must now be jointly examined in industrial fermentations to assess if these phenomena are correlated.

The presence of intracellular gas bubbles in both lager and ale fermenting strains also brings into question the current yeast flocculation paradigm. In the case of ale yeast, it is thought that these cells form loose flocs that trap evolved CO_2_ bubbles as they rise to the surface of the fermenting medium [30–32]. However, the work of Swart et al. [3] discovered that CO_2_ also becomes entrapped inside ale yeast cells which could also contribute to the buoyancy of the cells. It is now necessary to examine the effects of intracellular vs. intercellular CO_2_ bubbles on ale yeast flocculation. Furthermore, it remains to be elucidated why lager yeast settle to the bottom of a fermentation vessel and ale yeast float to the top when both cells accumulate intracellular CO_2_.

### Time-of-flight secondary ion mass spectrometry and confocal laser scanning microscopy

As with the NanoSAM results, the cell compositions detected by TOF-SIMS were different at the exterior and within the cell (Fig 5; Fig 6). Notably, with NanoSAM AES analysis, N and O were present at near equal levels during the targeted analysis (Fig 2B and 2D), but the TOF-SIMS analysis indicated the O^-^ ions were more intense than NH^-^ ions (Fig 5; Fig 6). With the NanoSAM AES analysis it was also not possible to detect P or S, but P^-^ and S^-^ could be detected with the TOF-SIMS (Fig 5; Fig 6). This discrepancy partially arises because the analysis area with the NanoSAM AES technique is more defined and specific (8 nm Auger resolution) than that achieved with TOF-SIMS (0.5–2 μm lateral resolution) [33]. Additionally, TOF-SIMS is a more sensitive surface analytical technique, with detection limits for most trace elements between 1E10 and 1E16 atoms/cc and in the parts per billion range [34]. However, TOF-SIMS does have the drawbacks that certain species are more readily ionized and easily detected, making quantitative analysis a challenge. To conduct quantitative analysis with TOF-SIMS, a reference sample with known concentrations of each ion is required so relative sensitivity factors can be calculated [35]. However, semi-quantitative analysis is still possible without a reference sample.

In the current yeast flocculation paradigm, hydrophobicity is thought to play an important controlling role in this phenomenon, but there is uncertainty whether cell surface proteins or lipids impart more of a hydrophobic effect [8,36]. Undoubtedly, future quantitative analysis of yeast cells using TOF-SIMS will help to increase our understanding of what classes of compounds, including 3-OH oxylipins, are implicated in cell surface hydrophobicity. As our lab works to achieve more quantitative elemental, molecular and 3-OH analysis of brewing yeast cells with TOF-SIMS, it is worthwhile noting that there is a disagreement in the literature around the pseudomolecular ion that predominates during negative mode TOF-SIMS analysis of hydroxy fatty acids.

Murase and Ohmori [37] reported that 2-OH 16:0 and 16-OH 16:0 formed the [M-H]^-^ pseudomoleular ions during negative mode TOF-SIMS analysis, meanwhile, Cersoy et al. [38] observed OH 16:0 and OH 18:0 as [M-3H]^-^ ions. To address this concern, we ran a 3-OH 10:0 standard (monoisotopic mass = 188.141245) on another ION-TOF TOF SIMS IV using a Bi^1+^ and Bi^3+^ source. With both the Bi^1+^ and Bi^3+^ source, it was the [M-H] ion that predominated with -61 and -50 ppm mass accuracy, respectively, when the monoisotopic mass was used as the theoretical value. While this is not as accurate as the maximal 8 ppm threshold used during precursor identification with a high resolution Orbitrap mass spectrometer (personal communication, Michal Surma, Researchgate), it is comparable to another study where up to -89 ppm mass accuracy was tolerated for fatty acid identification using negative mode TOF-SIMS analysis [39]. Therefore, TOF-SIMS holds substantial promise as tool to further examine cell composition and the 3-OH oxylipin signatures in brewing yeast.

Earlier immunofluorescence experiments examining 3-OH oxylipin distribution in *Dispodascopsis uninucleata* suggested a link between cell cycle, metabolism and 3-OH production as these oxygenated fatty acids were present in the sexual stage, but not during vegetative growth [16]. Immunofluorescence results of the SMA strain in YM vs. YPG media implied that 3-OH oxylipin production differed in fermenting and respiring cells, and thus with bubble production. For example, after only 24 h growth time, immunofluorescence trials indicated the respiring cells produced more 3-OH oxylipins than the fermenting cells (Fig 7B). While 3-OH oxylipins are believed to originate during β-oxidation or partial β-oxidation [40,41], the exact pathway which gives rise to these hydroxy fatty acids remains unknown. The results of this study indicate that the origin of 3-OH oxylipins in yeast should be better understood by studying the divergent lipid and fatty acid metabolisms in fermenting vs. respiring cells.

## Conclusions

In this study, the ability of Auger-architectomics to differentiate yeasts was demonstrated based on the previously published atomic concentrations of N in ascospores of *N*. *fulvescens* and the SMA strain of *S*. *pastorianus* examined here. TOF-SIMS depth profiling analysis also revealed that the relative proportions of C^-^ and O^-^ appeared to vary at the exterior and interior of the cell in the SMA strain and between fermentative or respirative growth metabolisms. Furthermore, the production of 3-OH oxlipins appeared to differ in fermenting and respiring cells, and thus with bubble production, based on investigations with immunofluorescence microscopy and CLSM. Therefore, this study has demonstrated differences in the composition and 3-OH oxylipin profile of bubble-filled and non bubble-filled SMA yeast cells and that the origin of 3-OH oxylipins in brewing yeast should be investigated by examining the divergent lipid and fatty acid metabolisms in fermenting vs. respiring cells. Finally, the presence of intracellular bubbles in bottom fermenting and top fermenting yeast strains has challenged the current brewing yeast flocculation paradigm. Further investigations on the influence of intracellular and intercellular CO_2_ on brewing yeast aggregation are now required.

## Supporting information

S1 Dataset Data used to generate Fig 2 and perform the associated statistics. (XLSX)

S2 Dataset Data used to generate Fig 5. (XLSX)

S3 Dataset Data used to generate Fig 6. (XLSX)
